# Surface electromyography analysis of mirror movements under unilateral movement in stroke patients: A retrospective study

**DOI:** 10.3389/fnhum.2022.1079596

**Published:** 2022-12-20

**Authors:** Jie Dai, Fangchao Wu, Jianhua Li, Mengjie Yu, Chen Liao, Yiqun Shou

**Affiliations:** ^1^Department of Rehabilitation Medicine, Sir Run Run Shaw Hospital, Zhejiang University School of Medicine, Hangzhou, Zhejiang, China; ^2^Department of Rehabilitation Medicine, Hospital of Zhejiang Chinese Armed Police Force, Hangzhou, Zhejiang, China; ^3^Department of Rehabilitation Medicine, The Third Hospital of Quzhou, Quzhou, Zhejiang, China

**Keywords:** surface electromyography, mirror movements, stroke, motor function, motor overflow

## Abstract

**Objective:**

Mirror movements (MMs) are common abnormal motor performance in patients with poststroke hemiparesis. The study aimed to utilize the Electromyography (EMG) characterization of MMs in stroke patients and explore the relationship between MMs and the motor function of affected limbs.

**Methods:**

Sixty patients with stroke who had used to undergo clinical assessment and surface Electromyography (sEMG) were selected in this study. We investigated the standardized net excitation (SNE) and overflow percentage (OF) as a measure of mirror activities on bilateral muscles of stroke patients.

**Results:**

In stroke patients, mirror activities occurred in both affected and unaffected muscles during maximal contractions. We found that OF at unilateral contraction on the affected side (UCA) was significantly greater than that at unilateral contraction on the unaffected side (UCU). Additionally, a negative correlation between OF at UCA and Brunnstrom stages on admission and discharge. However, there were no significant correlations between OF and disease duration, Barthel Index, or the degree of improvement in all clinical evaluations. We still found a positive correlation between SNE at UCA and the improvement of the Brunnstrom stage of the hand. But we could not find any significant correlation between SNE and other clinical evaluation scores.

**Conclusion:**

In conclusion, the study found mirror activities in both affected and unaffected muscles, confirming an asymmetry between them. Although the mechanisms are still unclear, we confirmed a significant correlation between MMs at UCA and the motor function of the affected upper extremity, which might provide further evidences for understanding MMs in stroke patients and a new research direction on evaluation for motor function and outcomes of stroke patients.

## 1 Introduction

Mirror movements (MMs) are involuntary movements occurring on one side of homologous muscles when unilateral voluntary movements are performed with the contralateral limb (Farmer, 2005). This phenomenon of movements has sometimes been documented as “global synkinesis (Hwang et al., 2005),” “motor overflow (Hoy et al., 2004),” or “contralateral irradiation (Hopf et al., 1974).” MMs can commonly be observed in healthy children under the age of ten or healthy adults who are involved in high-intense physical activities (Bodwell et al., 2003). Notably, MMs are very common in patients with poststroke hemiparesis. The incidence of MMs in stroke patients is about 54.8–70% (Lee et al., 2010), and they are usually observed in the upper extremities rather than in the leg or foot. Additionally, MMs appear in various stages of stroke and disappear gradually with the onset time (Chieffo et al., 2013; Ohtsuka et al., 2015; Ejaz et al., 2018). Several studies have found a correlation between the performance of MMs and the patient’s motor function (Kim et al., 2003; Hwang et al., 2005). Furthermore, MMs persist if the patient has a poor functional prognosis (Liu et al., 2021).

The evaluation of MMs is best achieved with surface Electromyography (sEMG). The EMG signals acquired by surface electrodes represent muscle activities in a region of interest. Importantly, previous studies showed that sEMG could be useful for assessing MMs after various neuropsychiatric disorders (Krampfl et al., 2004; Cincotta et al., 2006). Different from the traditional evaluation according to the methods of Woods and Teube, this technique can detect subtle activities in patients, which are referred to as mirror activities (Spagnolo et al., 2013). However, the clinical use of sEMG in assessing MMs after stroke is sporadic. One study has suggested a strong correlation between the change rate of mirror activities in the affected side after stroke and the patient’s upper extremity function score (Hwang et al., 2005). Furthermore, other research has found that the muscle activity of the unaffected limb is proportional to that of the mirror activity on the affected side (Chang et al., 2013). However, there are uncertainties about whether there was a correlation between the proportion and the motor function.

Considering that the proportion of bilateral muscle activities may better reflect the mutual influence between bilateral limbs (Chang et al., 2013), we hypothesized that, compared with the sEMG change rate, this parameter might better represent the motor function of the hemiplegic limb. Therefore, this study aimed to evaluate the mirror activities of bilateral upper limb muscles during unilateral movement with sEMG in patients with stroke and try to determine a correlation between them. Our findings may provide a better insight into MMs of stroke patients, and provide a new objective evaluation for motor functional outcomes after the onset of stroke. Moreover, further understanding of the interaction between affected limb and unaffected limb during movement after stroke, may also bring inspiration and enlightenment for innovative therapeutic interventions.

## 2 Materials and methods

### 2.1 Subjects

In this retrospective study, we reviewed the sEMG records conducted at Sir Run Run Shaw Hospital from June 2021 to May 2022. Among patients who underwent sEMG, those with brain infarction confirmed by magnetic resonance imaging were selected. Of the 66 patients with brain infarction initially selected, 64 met the following inclusion criteria: Patients hospitalized for ischemic stroke, patients who have completed the sEMG assessment and functional scale assessment, including Brunnstrom Stages of Motor Recovery Scale (BR) and Barthel Index (BI), within 1 week after hospitalization. Regarding the exclusion criteria, patients with a history of other diseases affecting the motor function of the upper extremities were excluded. Likewise, patients with poor sEMG data or incomplete clinical data were excluded from the research. None of the patients had any limitations in the passive range of motion. Finally, there were 60 patients involved in our study. The medical history of all the patients was taken from the patient medical record. The motor recovery status was between stages I and VI in the BR.

The experimental protocol was approved by the Ethics committee of Sir Run Run Shaw Hospital, Zhejiang University School of Medicine (2022-534-01).

### 2.2 Clinical evaluation

All 60 patients had finished the BI of Activities of Daily Living (ADL) for independence in mobility and personal care (range 0–20) and a BR stage for motor recovery (range I–VI). Both were completed 1 week after hospitalization and on the day of discharge. Because of the higher patient compliance requirements, 41 patients had an arm motor score on the Fugl-Meyer Assessment Scale (FMA) 1 week after hospitalization, and only 24 patients had finished the same assessment before discharge. The same trained physical therapist performed all clinical assessments to avoid individual error. Furthermore, the evaluator was blinded to the content and purpose of the research.

### 2.3 sEMG

#### 2.3.1 Apparatus

**FIGURE 1:**
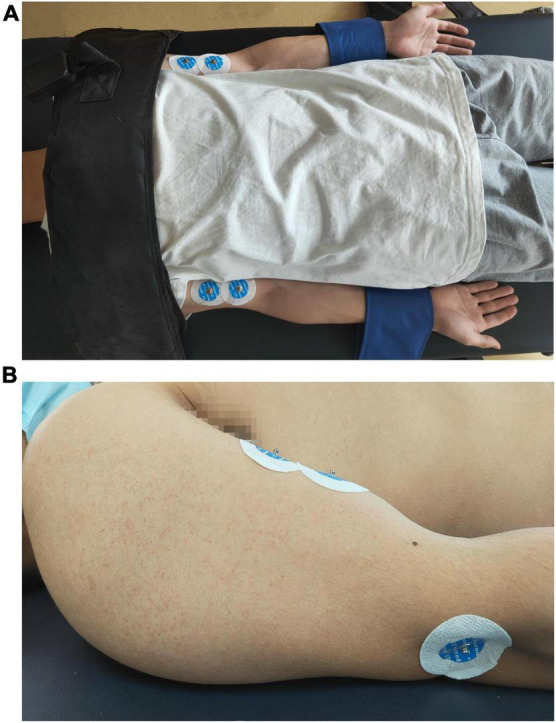
Positions of the surface electromyography electrodes. **(A)** A photo of a volunteer to show the position of the body, the fixation belts and the location of the electrodes on bilateral upper extremities. **(B)** A photo of a volunteer to show the lateral side of the location of the electrodes on upper extremity.

Subjects were asked to lay on a height-adjustable bed, with two legs straight and two feet close to the bed pedal. Both upper limbs were symmetrically close to the trunk, the shoulders and elbows were fully extended, and the forearms/wrists were maintained in a neutral position. Additional stabilization straps were applied to the distal forearms to prevent the flexion of the elbow joint, and a harness with a shoulder strap secured the trunk to the back support to avoid compensatory axial movement or shoulder protraction and retraction force generated by the joint’s movement during the task. Bipolar surface EMG electrodes (Ag/AgCl, diameter = 1.5 cm, LT-301) were placed over the muscle bellies of biceps brachii bilaterally, and a reference electrode was placed at the lateral humeral epicondyle on the left side (see Figure 1), as described in a recent study (Chang et al., 2013).

The sEMG was recorded using a medstra biomedical acquisition system (Model AMT-4, common mode rejection ratio of 130 dB at 60 Hz, input impedance of 10 GΩ). EMG signals were digitized at 1,000 Hz with a 12-bit A/D converter (PCI 6024E, Shaoxing United Medical Instruments, Zhejiang, China). High-and low-frequency filters were set at 500 Hz and 20 Hz. All the data was collected with custom LabVIEW software, processed by Megawin3.1 (Math Works, Natick, MA), and saved for offline analysis.

#### 2.3.2 Tasks

The enrolled subjects were not aware of the focus of our research interests to avoid any participant bias or awareness that might affect the movements. They performed a set of voluntary isometric muscle contractions with visual feedback in a fixed order: (1) Unilateral contraction tasks on the affected side (UCA) and (2) unilateral contraction tasks on the unaffected side (UCU). During the task, the subject was asked to perform voluntary isometric muscle contractions with maximal force and relax the contralateral limb. In each set, three contractions should be completed, each contraction lasted 5 s, and the interval was 5 s. A root-mean-square (RMS) voltmeter provided visual feedback on EMG activity to enable subjects to maintain a steady muscle contraction (Mayston et al., 1999). The reduction of RMS should be controlled within 10% of the maximum. To reduce individual error, the same physician performed the assessment, blinded to the content and purpose of the research.

#### 2.3.3 Data processing

Resting baseline EMG of the resting limb was obtained first before movement of the contralateral limb. Average amplitudes of EMG (AEMG) and RMS in the 3-s segment were calculated through the absolute values of EMG signals. The activity patterns of sEMG for the bilateral muscles (both active and resting limbs) were subsequently gained during two tasks. A period of 3 s of EMG signals was used due to stroke patients being too weak to maintain the steady force for a long time (Lodha et al., 2012). AEMG and RMS in a 3-s segment were calculated for both limbs. Finally, the mean AEMG and RMS value was determined by averaging the values of the 3 trials for each task.

The standardized net excitation (SNE) was defined as the irradiation of the resting muscle attributed to the contraction of the contralateral homologous muscle. As described in a recent study, the SNE of an irradiated muscle was defined by subtracting the value for irradiated EMG from its baseline activity and then normalizing the value of the baseline activity. The SNE was calculated in the following equations (Hwang et al., 2005):


$${\text{SNE}}_{\text{AEMG}}=\frac{{\text{AEMG}}_{\text{rest}}-{\text{AEMG}}_{\text{baseline}}}{{\text{AEMG}}_{\text{baseline}}};$$

$${\text{SNE}}_{\text{RMS}}=\frac{{\text{RMS}}_{\text{rest}}-{\text{RMS}}_{\text{baseline}}}{{\text{RMS}}_{\text{baseline}}}.$$

The overflow of EMG activity recorded in the resting limb compared to the amount of EMG activity in the active limb was measured. An overflow percentage (OF) was calculated using the EMG value of the resting limb and the EMG value of the contracting limb following equation (Chang et al., 2013):


$${\text{OF}}_{\text{AEMG}}=\frac{{\text{AEMG}}_{\text{rest}}}{{\text{AEMG}}_{\text{contract}}};$$

$${\text{OF}}_{\text{RMS}}=\frac{{\text{RMS}}_{\text{rest}}}{{\text{RMS}}_{\text{contract}}}.$$

### 2.4 Statistical analysis

The Chi-square test were used to compare male-to-female ratio and infarction side of the LFG and HFG. The Mann–Whitney *U*-test was applied to determine differences in sEMG parameters between the affected and unaffected muscles of patients with stroke. For the correlation of sEMG parameters with the clinical assessment and disease duration, the Spearman rank correlation coefficient was used. According to the BR score of the affected arm, the patients were divided into two groups, High Function Group (HFG, *n* = 31, *BR* > 3) and Low Function Group (LFG, *n* = 29, *BR* ≤ 3), to further clarify the correlation between EMG parameters and clinical function. Furthermore, the Mann–Whitney *U*-test was used to determine differences in sEMG parameters between the two groups. All data are displayed as Means ± Standard deviation. The level of significance was set at 0.05. In order to show the difference more clearly, all the data used in the figures were logarithmically converted. Finally, all statistical analyses were completed using SPSS version 24.0 (IBM Corporation, Armonk, NY).

## 3 Results

### 3.1 Demographics

Sixty stroke patients were included in this study. The average age, male-to-female ratio, infarction side, disease duration, and clinical assessment scores are summarized in Table 1. There were no significant differences in average age, male-to-female ratio, and infarction side between the two groups (*p* > 0.05). The disease duration of LFG is significantly longer than that of HFG (*p* < 0.05).

**TABLE 1:** Characteristics of subjects and groups.

Characteristic	Stroke patients (*n* = 60)	HFG (*n* = 31)	LFG (*n* = 29)
Age, years (y)	60.47 ± 14.58	61.61 ± 15.92	59.24 ± 13.17
Gender, male: female, *n*	49:11	25:6	24:5
Infarction side, right: left, *n*	28:32	18:13	10:19
Disease duration, days (d)	49.75 ± 72.61	40.00 ± 45.68	60.17 ± 93.04*

HFG, high function group; LFG, low function group; *p < 0.05.

### 3.2 sEMG parameters

**FIGURE 2:**
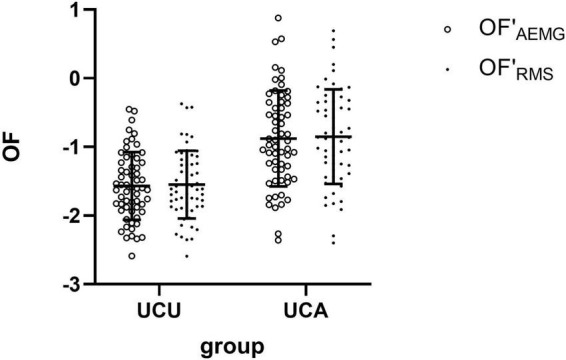
The comparison (means with standard deviations in parentheses) of the OF′_AEMG_ and OF′_RMS_ of the bilateral biceps brachii during unilateral contraction tasks. OF′_AEMG_ = Log (OF_AEMG_), OF′_RMS_ = Log (OF_RMS_).

**FIGURE 3:**
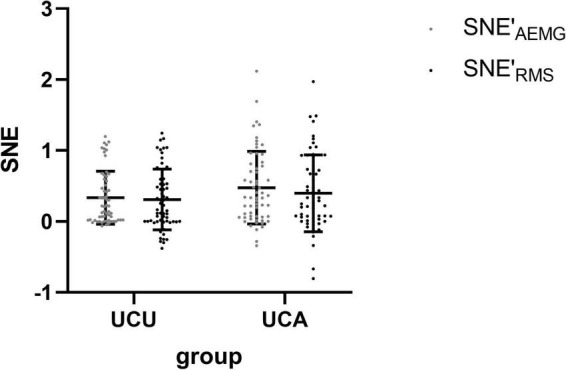
The comparison (means with standard deviations in parentheses) of the SNE′_AEMG_ and SNE′_RMS_ of the bilateral biceps brachii during unilateral contraction tasks. SNE′_AEMG_ = Log (SNE_AEMG_ + 1), OF′_RMS_ = Log (OF_RMS_ + 1).

Table 2 shows the sEMG parameters of bilateral biceps brachii of patients with stroke. There were significant differences in the OF_*AEMG*_ and OF_*RMS*_ during unilateral movement between the unaffected and affected extremities (*p* < 0.01). OF (including OF_*AEMG*_ and OF_*RMS*_) was significantly greater during unilateral tasks of the affected limb than on the unaffected side (see Figure 2). However, there were no significant differences in SNE_*AEMG*_ or SNE_*RMS*_ (*p* > 0.05), although SNE (including SNE_*AEMG*_ and SNE_*RMS*_) at unilateral tasks of the affected limb was typically greater than that of the unaffected limb (see Figure 3).

**TABLE 2:** Surface Electromyography parameters of bilateral biceps brachii of patients with stroke.

	UCU	UCA
SNE_AEMG_	2.325 ± 3.719	6.504 ± 18.143
SNE_RMS_	2.423 ± 3.989	5.229 ± 13.286
OF_AEMG_	0.052 ± 0.072	0.486 ± 1.150**
OF_RMS_	0.056 ± 0.086	0.446 ± 0.865**

SNE, standardized net excitation; OF: overflow percentage; UCU, unilateral contraction of unaffected side; UCA, unilateral contraction of affected side; **p < 0.01.

### 3.3 sEMG parameters and clinical assessment

Tables 3, 4 display the correlations between sEMG parameters during unilateral maximal isometric flexion tasks and disease duration, BI score, and BR stages of patients with stroke. There was a negative correlation between OF at the affected limb contraction task and BR stages on admission. Additionally, there was a negative correlation between OF at the affected limb contraction task and BR stages of upper extremity and hand at discharge. There were no significant correlations between OF and disease duration, BI score, or the degree of improvement in all clinical evaluations. However, there was a positive correlation between SNE at the affected limb contraction task and the improvement of the BR stage of the hand. Furthermore, there were no significant correlations between SNE and any other clinical evaluation scores.

**TABLE 3:** Spearman correlation coefficients (ρ) for OF and clinical assessments.

OF_AEMG_					OF_RMS_			
*n* = 60	UCU		UCA		UCU		UCA	
	*ρ*	*P*−value	*ρ*	*P*-value	*ρ*	*P*−value	*ρ*	*P*-value
*DD*	0.068	0.605	0.179	0.171	0.094	0.476	0.210	0.108
*BR*−*UE*^*a*^	0.052	0.691	–0.378	0.003**	0.061	0.642	–0.371	0.003**
*BR*−*H*^*a*^	0.111	0.399	–0.407	0.001**	0.137	0.296	–0.403	0.001**
*BR*−*L*^*a*^	-0.117	0.372	–0.318	0.013*	-0.105	0.425	–0.309	0.016*
*BI* ^*a*^	-0.197	0.131	–0.235	0.071	-0.189	0.149	–0.225	0.084
*BR*−*UE*^*d*^	0.056	0.692	–0.407	0.001**	0.047	0.719	–0.416	0.001**
*BR*−*H*^*d*^	0.089	0.498	–0.291	0.024*	0.095	0.472	–0.301	0.019*
*BR*−*L*^*d*^	-0.033	0.804	–0.239	0.066	-0.032	0.807	–0.242	0.063
*BI* ^*d*^	-0.208	0.110	–0.239	0.065	-0.215	0.099	–0.240	0.065
*BR*−*UE*^*c*^	-0.031	0.815	–0.014	0.915	-0.053	0.685	–0.036	0.784
*BR*−*H*^*c*^	-0.097	0.460	0.223	0.087	-0.130	0.321	0.214	0.100
*BR*−*L*^*c*^	0.128	0.329	0.263	0.042*	0.101	0.442	0.245	0.060
*BI* ^*c*^	0.145	0.268	0.144	0.272	0.115	0.380	0.125	0.340

DD, disease duration; BR-UE, Brunnstrom stage of upper extremity (shoulder and elbow); BR-H, Brunnstrom stage of hand; BR-L, Brunnstrom stage of lower extremity; BI, Barthel index; ^a^assessment at admission; ^d^assessment at discharge; ^c^the improvement of assessment score. *p < 0.05; **p < 0.01.

**TABLE 4:** Spearman correlation coefficients (ρ) for SNE and clinical assessments.

	SNE_AEMG_				SNE_RMS_			
*n* = 60	UCU		UCA		UCU		UCA	
	*ρ*	*P*-value	*ρ*	*P*-value	*ρ*	*P*−value	*ρ*	*P*-value
*DD*	0.127	0.334	–0.017	0.895	0.066	0.616	–0.096	0.468
*BR*−*UE*^*a*^	-0.026	0.844	0.017	0.899	0.025	0.850	–0.162	0.217
*BR*−*H*^*a*^	0.093	0.478	–0.024	0.854	0.133	0.312	–0.103	0.434
*BR*−*L*^*a*^	0.059	0.655	0.091	0.488	0.074	0.573	–0.083	0.529
*BI* ^*a*^	-0.006	0.964	0.118	0.370	0.026	0.844	0.013	0.920
*BR*−*UE*^*d*^	-0.035	0.790	0.058	0.661	0.020	0.879	–0.064	0.624
*BR*−*H*^*d*^	0.115	0.381	0.175	0.181	0.162	0.215	0.090	0.492
*BR*−*L*^*d*^	0.141	0.281	0.145	0.269	0.139	0.289	0.004	0.979
*BI* ^*d*^	0.007	0.957	0.183	0.162	0.051	0.698	0.089	0.499
*BR*−*UE*^*c*^	-0.019	0.888	0.064	0.630	-0.041	0.758	0.190	0.146
*BR*−*H*^*c*^	-0.071	0.590	0.330	0.010*	-0.054	0.680	0.296	0.022*
*BR*−*L*^*c*^	0.033	0.801	0.028	0.831	0.006	0.966	0.160	0.222
*BI* ^*c*^	0.093	0.478	0.068	0.607	0.091	0.490	0.096	0.466

DD, disease duration; BR-UE, Brunnstrom stage of upper extremity (shoulder and elbow); BR-H, Brunnstrom stage of hand; BR-L, Brunnstrom stage of lower extremity; BI, Barthel index; ^a^assessment at admission; ^d^assessment at discharge; ^c^the improvement of assessment score. *p < 0.05.

Since fewer patients had finished the FMA assessment of the upper extremity, we separately compared the correlation between OF/SNE and FMA (Table 5). We identified a negative correlation between OF at the affected limb contraction task and FMA scores at admission and discharge. At the same time, there was no significant correlation between SNE and FMA scores.

**TABLE 5:** Spearman correlation coefficients (ρ) for OF/SNE and FMA.

	FMA^a^ (*n* = 41)		FMA^d^ (*n* = 24)		FMA^c^ (*n* = 23)		
	*ρ*	*P*-value	*ρ*	*P*-value	*ρ*	*P*-value	
OF_AEMG_	*UCU*	–0.073	0.652	–0.098	0.649	-0.200	0.361
	*UCA*	–0.371	0.017*	–0.458	0.024*	-0.234	0.282
OF_RMS_	*UCU*	–0.079	0.623	–0.071	0.740	-0.184	0.400
	*UCA*	–0.366	0.019*	–0.450	0.028*	-0.248	0.253
SNE_AEMG_	*UCU*	0.106	0.508	0.020	0.926	-0.116	0.597
	*UCA*	–0.004	0.980	0.095	0.659	0.190	0.386
SNE_RMS_	*UCU*	0.113	0.482	0.030	0.891	0.015	0.946
	*UCA*	–0.172	0.282	–0.094	0.664	0.195	0.372

FMA, Fugl-Meyer Assessment Scale of upper extremity. ^a^assessment at admission; ^d^assessment at discharge; ^c^the improvement of assessment score. *p < 0.05.

To confirm the correlation between sEMG parameters and clinical assessment, we divided the patients into HFG and LFG and compared the sEMG parameters between the two groups. Table 6 shows that OF at UCA was significantly greater in LFG than in HFG (*p* < 0.01). As expected, the two groups had no significant difference in OF at UCU and SNE at both tasks.

**TABLE 6:** Surface electromyography parameters of patients with stroke between two groups.

		LFG (*n* = 29)	HFG (*n* = 31)
OF_AEMG_	UCU	0.044 ± 0.065	0.059 ± 0.078
	UCA	0.743 ± 1.491	0.245 ± 0.634**
OF_RMS_	UCU	0.048 ± 0.080	0.064 ± 0.092
	UCA	0.648 ± 1.073	0.256 ± 0.566**
SNE_AEMG_	UCU	2.492 ± 4.076	2.168 ± 3.413
	UCA	9.020 ± 25.273	4.151 ± 6.276
SNE_RMS_	UCU	2.627 ± 4.298	2.231 ± 3.737
	UCA	7.411 ± 18.023	3.188 ± 5.864

LFG, low function group; HFG, high function group. **p < 0.01.

## 4 Discussion

In this study, stroke subjects performed voluntary isometric muscle contractions at maximal levels with the affected and unaffected limbs unilaterally. We chose two sEMG parameters, AEMG and RMS, to examine the electrical activity of the target muscles. We found no significant difference in results between the two parameters in all the analyses. Although the absolute values of RMS and AEMG were different, their results were completely consistent on the comparison between the affected and unaffected sides and the high and low function groups, as well as the correlation with motor function. Notably, both AEMG and RMS are important in estimating the change of sEMG signals (Felici et al., 1997; Potvin and Bent, 1997). Additionally, the amplitude change underestimates the associated change in motor unit activity underlying muscle force modulation (Farina et al., 2004). In past research, people always used RMS to express the electrical activities of muscles (Hwang et al., 2005; Chang et al., 2013; Liaw et al., 2021). However, according to our findings, AEMG and RMS may have the same value in expressing the sEMG characteristics of MM.

As expected, we found mirror activities in both affected and unaffected muscles during maximal contractions with the help of sEMG techniques. Previously, it’s been believed that MMs always appeared predominantly in the distal upper limb muscles, especially the hands, and occurred on the unaffected side when patients move the paretic hand (Cox et al., 2012). Notably, only a few investigations proved that MMs could occur in other parts of the limb (Hwang et al., 2005), even the leg or foot (Tubbs et al., 2004), and bilateral limbs (Nelles et al., 1998; Chang et al., 2013). In contrast, our findings suggest that MMs might appear bilaterally in the proximal upper limb muscles, although we couldn’t observe the movements directly. Considering the complexity of finger movement, the voluntary contraction of the proximal flexor muscle is much easier for stroke patients. Therefore, it could be a better choice in MMs research in the future.

We also found a significant difference between sides in the electrical activity of muscles at two tasks. Notably, the OF at UCA was significantly higher than that at UCU. Although there was no significant difference in RE, an apparent trend was observed that SNE at UCA was higher than that at UCU. Therefore, we confirmed the asymmetry in motor overflow between affected and unaffected elbow flexion in stroke subjects, which was also observed by Chang et al. (2013). Compared with SNE, OF could show such asymmetry more effectively. People once found that the MM was bilaterally symmetric in normal adults, and both limbs’ clinical presentation had no difference (Armatas et al., 1994). However, the injury of the brain poststroke might destroy the symmetry. Because of the weakness of the affected muscle at maximal voluntary contraction task, more activation of motor areas of both hemispheres might be induced. Consequently, activating the contralesional hemisphere might cause more overflow to the unaffected side (Cleland and Madhavan, 2022). Previous neuroimaging studies have reported increased activity in the non-lesioned sensorimotor cortex poststroke during active movements of the affected limb (Wittenberg et al., 2000; Auriat et al., 2015) and demonstrated a close relationship between MMs and the unaffected motor cortex activation (Kim et al., 2003), consistent with our results.

There are two possible explanations for the phenomena above. One is the reduction of interhemispheric inhibition (IHI). In healthy people, activating the motor area in one hemisphere causes inhibition of the homologous cortical area in the contralateral hemisphere, which is called IHI (Archontides and Fazey, 1993). After a stroke, the lesion of one hemisphere might decrease the inhibition, so more activation of the contralateral hemisphere might be induced (Caronni et al., 2016), which causes more motor flow to the unaffected side. The other reason may be the unmasking of ipsilateral corticospinal projections. In previous studies, when a single pulse of TMS was applied to an inactive region of the primary motor cortex (M1), bilateral motor-evoked potentials (MEPs) could be detected in the target muscles (Etoh et al., 2010; Chieffo et al., 2013). This phenomenon might indicate the existence of ipsilateral corticospinal projections. Our findings assumed that after stroke, the contralesional motor cortex was activated to promote the motor function of the affected limb at maximal contraction tasks *via* ipsilateral corticospinal tracts.

Mirror movements may reflect the motor function of patients with stroke. Conversely, people found that the location of MMs might be related to the motor function of the paretic hand. Notably, MMs in the unaffected hand had been associated with a significantly worse motor function (Nelles et al., 1998; Jiang et al., 2020). Hwang et al. (2005) found that the relationship between motor function and MMs at UCU in the proximal muscles was rather obvious than between motor function and MMs in the distal muscles. Thus, we compared the correlations between sEMG parameters of proximal flexor muscle and clinical motor function assessment scores and found a significantly negative correlation between OF at UCA and BR stages/FMA scores of upper extremities. Furthermore, we found that OF was not only related to the motor function of the upper limb at admission but also related to the function at discharge. Thus, the functional prognosis of patients could be predicted with such sEMG parameters to some extent. There was also a significant positive correlation between SNE at UCA and improvement of BR stages of hands, which meant that RE might not directly reflect the upper extremity function of patients. Still, it could predict the recovery potential of the upper extremity. We did not find a positive correlation between SNE at UCU and BR stages and FMA scores, which was discovered by Hwang et al. (2005) likely due to different methods of EMG detection. In their research, they did not control the movement pattern of the affected limb. Therefore, the EMG signal of the affected limb in their experiment was not from the isometric muscle contraction.

The explanation for the correlation between MMs and motor function is still unclear. In the IHI hypothesis, the increased inhibition from the contralesional hemisphere may play an important role in hindering the recovery of motor function for stroke patients (Takeuchi and Izumi, 2012). Notably, OF is the ratio of the overflow of contralesional muscle to the sEMG value of the affected muscle contraction (Chang et al., 2013). Compared with SNE, it can better reflect the over-activation of the contralesional cortex and its inhibition of the lesioned cortex. Our study found a significant negative correlation between OF and motor function of the affected side during muscle contraction, which also suggested that interhemispheric inhibition imbalance might be an essential factor leading to the motor dysfunction of stroke patients.

In our research, we also found that SNE was not related to the motor function of patients but had a positive relationship with the improvement of motor function, indicating that the activation of the contralateral cortex may be related to the recovery potential of motor function of the affected side. Importantly, this phenomenon may be because of ipsilateral projection. According to the hypothesis of ipsilateral projection, the inhibition of lesioned motor cortex or the loss of contralateral corticospinal tracts might cause the decreased neural drive to the affected muscle. At this time, the contralesional cortex would be activated to improve the motor function of the affected side *via* ipsilateral corticospinal tracts (Bestmann et al., 2010; Rehme et al., 2011). Therefore, this might be an important reason for motor function recovery after stroke (Alawieh et al., 2017). Notably, our findings show that the greater the degree of cortical activation, the greater the potential for functional recovery.

Another unexpected finding was that the MMs of the proximal muscles are not only negatively correlated with the motor function of the proximal limb but also negatively correlated with the motor function of the distal limb or even the lower limb. This finding might be best explained from the perspective of neuroanatomy. As acknowledged in neuroanatomy, proximal muscles have more bilateral interneurons and transcallosal projections at both cortical and spinal levels compared to distal muscles (Rouiller et al., 1994; Jankowska et al., 2005). Therefore, compared with distal muscles, the change in motor activity from proximal muscles could more significantly reflect the neural interactions between the two hemispheres (Aune et al., 2020). Since such a change of interhemispheric interactions affects not only the motor function of affected proximal muscles but also that of affected distal muscles and lower extremities (Takeuchi and Izumi, 2012), our findings above seemed acceptable. However, it was difficult to explain why SNE at UCA was only related to the recovery potential of hand function but not the recovery potential of proximal muscles. One possible explanation is that there might be two different mechanisms for the functional recovery of proximal and distal extremities (Jung et al., 2002; Hwang et al., 2005), and the changes of MMs are only related to the functional recovery of distal extremities. However, neither mechanism has been proven directly since now.

To further investigate the correlation between sEMG parameters and motor function of the upper extremity, we divided all the patients into two groups, according to the BR grade of arms. We found out that, compared with patients from LFG, OF of patients from HFG at UCA was significantly reduced and more similar to OF at UCU. As we mentioned above, a symmetry in mirror movement of both upper limbs in the Healthy Population might be destroyed in patients with stroke. Based on our findings, we believed that the more serious the asymmetry of the mirror movements, the worse the patient’s motor function.

Although there are two acceptable hypotheses, the pathological mechanism of MMs is still unclear. Furthermore, there is a lack of reliable evidence to explain the relationship between MMs and motor function. Wittstock et al. (2020) used diffusion tensor imaging technology to elucidate structural alterations of callosal integrity in ALS patients with MMs. However, they failed to show microstructural changes accompanying mirror movements and disturbed transcallosal inhibition (Wittstock et al., 2020). In the future, more neuroimaging and neurophysiological techniques are needed to explain the pathological mechanism of MMs and the relationship between MMs and the motor function of the affected limbs.

As a retrospective study, several limitations of this study should be noted. First, we lacked a control group including patients without stroke, so only comparisons within the group could be tested. Additionally, the research results from other scientists for patients without stroke were directly adopted in this report, and this approach may be controversial because of the different study methods. In the future, we need to set up a proper control group and directly find out the characteristic changes of patients’ MMs by comparing groups. In addition, the location of the lesion may also affect the performance of MMs. In this article, we had not strictly limited the cause of stroke and the lesion location. Therefore, we cannot explore the impact of such factors on MMs. Therefore, this may be a direction of future research.

## 5 Conclusion

In conclusion, our study replicates and extends previous findings and has made a more detailed analysis of the EMG characterization of MMs in stroke patients. We have found mirror activities in both affected and unaffected muscles, confirming an asymmetry between them. Although the mechanisms are still unclear, we also found a significant correlation between MMs at UCA and the motor function of the affected upper extremity. Therefore, this study provides further evidence for understanding MMs in stroke patients and provides a new research direction on evaluation for motor function and outcome. Of course, further studies in more extensive and more homogeneous cohorts are still needed for validation.

## Data availability statement

The raw data supporting the conclusions of this article will be made available by the authors, without undue reservation.

## Ethics statement

The studies involving human participants were reviewed and approved by the Ethics committee of Sir Run Run Shaw Hospital, Zhejiang University School of Medicine. Written informed consent for participation was not required for this study in accordance with the national legislation and the institutional requirements. Written informed consent was obtained from the individual(s) for the publication of any potentially identifiable images or data included in this article.

## Author contributions

JD: conception, design, drafting of manuscript, analysis, and final approval of manuscript. FW: conception, design, interpretation of data, revision of manuscript, and final approval of manuscript. JL: conception, revision of manuscript, and final approval of manuscript. MY and CL: acquisition of data and final approval of manuscript. YS: concept, design, revision of manuscript, and final approval of manuscript. All authors contributed to the article and approved the submitted version.
